# Dissipation Behavior and Risk Assessment of Three Pesticide Residues Under Combined Application in Greenhouse-Grown Cabbage

**DOI:** 10.3390/foods14173006

**Published:** 2025-08-28

**Authors:** Caixia Sun, Liping Chen, Yuhong Liu, Weiran Zheng, Yumei Hua, Qiaoyan Zhang

**Affiliations:** 1 Institute of Agro-Product Safety and Nutrition, Zhejiang Academy of Agricultural Sciences, Hangzhou 310021, China; liuyh2028@126.com (Y.L.); zhengwr@zaas.ac.cn (W.Z.);; 2 Huzhou Agricultural Science and Technology Development Center, Huzhou 313009, China; chenliping20042000@163.com; 3 College of Resources and Environment, Huazhong Agricultural University, Wuhan 430072, China

**Keywords:** cabbage, pesticide, dissipation behavior, risk assessment, greenhouse

## Abstract

Field residue trials were conducted in greenhouse-grown cabbage at both recommended and double dosages to evaluate the degradation dynamics and dietary risks of three pesticides (azoxystrobin, thiamethoxam, and carbendazim). In this study, a quick, easy, cheap, effective, rugged, and safe (QuEChERS) method combined with liquid chromatography tandem mass spectrometry was developed to measure the residues of three pesticides in cabbage. The mean recoveries of three pesticides in cabbage were 82.5–104.2%, with relative standard deviations of 2.1–5.2%, meeting the requirements of residual analysis. Based on first-order kinetics, the half-lives of the three pesticides in cabbage were 11.55–33.00 d under field conditions. The health risks associated with three pesticides in cabbage were evaluated using the risk quotient (RQ) method and the EFSA PRIMo 3.1 model. In the final residue experiment, the dietary exposure risks of azoxystrobin and thiamethoxam were all acceptable for children and adults regardless of the dosage or pre-harvest intervals, with the risk quotient (RQ) ranging from 0.040 to 0.363 and 0.022 to 0.417, respectively. However, carbendazim intake posed unacceptable health risks for consumers, with RQ significantly exceeding 1. The EFSA PRIMo 3.1 model also indicated high %ADI values for carbendazim, consistent with the RQ results. Given the significant residual risk associated with carbendazim in cabbage, limiting its use on this crop is recommended.

## 1. Introduction

Pesticides serve as crucial tools in agricultural production for controlling pests and diseases and are characterized by their high efficiency, rapid action, and ease of application. Since 1995, China has maintained a sustained growth trajectory in pesticide utilization, becoming one of the largest consumers of agricultural pesticides globally [1]. According to the Food and Agriculture Organization of the United Nations, China’s pesticide consumption reached 235.76 kilotons in 2022, with an average application intensity of 1.83 kg/ha [2]. Greenhouse cultivation is widely used in modern agriculture, especially vegetable production. It enables agricultural production to break through the constraints of seasons and regions, generating more employment opportunities and higher economic benefits [3]. The cultivated area of facility vegetables increased from 5300 ha in 1978 to approximately 4,000,000 ha in 2018, with a 755-fold increase in 40 years [4]. However, due to increased planting density, pesticide application is inevitable for pest and disease control in greenhouses, elevating the risk of excessive toxic and hazardous substance residues. In recent years, improper pesticide use has posed significant potential threats to the safety and quality of greenhouse-grown vegetables.

Cabbage (*Brassica oleracea*), a cruciferous vegetable, has become a dietary staple across Chinese households due to its culinary versatility and high adaptability. As it is rich in dietary fiber, vitamins, minerals, and beta-carotene and low in calories, it has been recognized by the World Health Organization as one of the best vegetables and is now cultivated worldwide [5,6]. *Brassica oleracea* vegetables exert cytoprotective effects against oxidative stress-induced tissue damage and demonstrate antimicrobial activity against bacterial and fungal pathogens [7,8]. Epidemiological studies have indicated that their consumption may reduce the risk of cardiovascular disease and cancer [9]. Major diseases affecting cabbage include downy mildew, black rot, viral diseases, brown rot, Sclerotinia rot, brown spot, Fusarium wilt, and soft rot, and the main pests include diamondback moths, noctuid moths, and cabbage worms. Registration data from the China Pesticide Information Network indicate that cabbage emerged as a focal crop for pesticide formulations in 2024, with 76 pesticide products registered for use against five major targets: cabbage worms, flea beetles, beet armyworms, diamondback moths, and aphids.

Azoxystrobin, a methoxyacrylate fungicide, inhibits mitochondrial respiration by blocking electron transfer between cytochrome b and cytochrome c1 in Complex III of the mitochondrial electron transport chain, thereby suppressing mycelial growth and spore germination [10]. Due to vegetables’ susceptibility to fungal diseases, extensive fungicide use is common in vegetable production, with azoxystrobin being the most widely used. Studies have shown that azoxystrobin is the most frequently detected fungicide in leafy vegetables sampled in South Korea, with a detection rate of 17.8% [11]. Thiamethoxam, a neonicotinoid insecticide, has high efficacy, broad-spectrum activity, and low mammalian toxicity and acts as a stomach poison, contact toxin, and systemic agent, having rapid action and long-lasting residual effects. Thiamethoxam demonstrates excellent control against sap-sucking pests, such as whiteflies, aphids, planthoppers, and thrips [12]. It is currently registered for use on various vegetable crops, such as solanaceous vegetables, leafy greens, and root vegetables, in China. Carbendazim, a cost-effective broad-spectrum systemic benzimidazole fungicide, disrupts pathogen DNA synthesis and inhibits spindle formation, thereby inducing apoptosis. It effectively controls fungal diseases, such as mold, spot diseases, powdery mildew, scorch, rot, and Fusarium wilt [13,14]. With a soil half-life exceeding 12 months, carbendazim exhibits environmental persistence and stability [15,16]. Due to the emergence of resistance, it is often applied at high frequencies and dosages, which may lead to residual contamination of the environment. Consequently, extensive use may result in its accumulation in ecosystems and food chains [17].

The selection of carbendazim, azoxystrobin, and thiamethoxam for this study is fundamentally justified by their widespread co-application in cabbage production, despite regulatory particularities. Field surveys confirm that growers routinely adopt this combination to achieve broad-spectrum control against cabbage’s complex pest-disease system (e.g., simultaneously managing downy mildew/brown spot with fungicides and flea beetles/aphids with insecticides). This practice aligns with China’s extensively implemented “Green Food—Guideline for Application of Pesticide (NY/T 393-2020)” [18], which explicitly endorses these compounds for controlling downy mildew, brown spot, and flea beetles in vegetables. However, this co-application operates within a significant regulatory gap: carbendazim and azoxystrobin currently lack official registration for cabbage or other cruciferous vegetables in China. This paradoxical situation—where guideline-recommended practices involve unregistered pesticides—creates substantial uncertainties regarding residue dynamics, dietary risks, and regulatory compliance. Consequently, investigating the dissipation dynamics and risk profiles of this practically relevant mixture under authentic greenhouse conditions is imperative. This study conducted field residue trials on cabbage in greenhouses, applying the recommended dose and double dose, with sampling conducted within 0–28 days. Through residue analysis and risk assessment, this study quantified the impact of the three-pesticide mixture on cabbage residues and provided baseline residue–risk data for this practically applied combination under greenhouse conditions. These results serve as a reference for the risk management of three pesticides. Consequently, the work advances understanding of the environmental fate and food safety implications of a widely used pesticide mixture in cabbage, with direct relevance to (1) regulatory science—closing data gaps for unregistered uses and supporting MRL establishment; (2) food safety enhancement—refining multi-residue risk assessment; and (3) sustainable agriculture—guiding rational pesticide use and reducing residue risks. However, given that trials were restricted to one season and one greenhouse region, these recommendations require validation under open-field and multi-regional conditions before broader adoption.

## 2. Materials and Methods

### 2.1. Chemicals and Reagents

Methanol and acetonitrile (chromatography grade) were purchased from Merck KGaA (Darmstadt, Germany). Ammonium formate (chromatography grade) was supplied by Tedia Co., Ltd. (Fairfield, OH, USA). Primary secondary amine (PSA) and C_18_ adsorbents were obtained from Tianjin Bonna Agela Technology Co., Ltd. (Tianjin, China). Anhydrous magnesium sulfate and sodium chloride were purchased from Shanghai Lingfeng Chemical Reagent Co., Ltd. (Shanghai, China). The azoxystrobin standard (98.0% purity) was provided by the Shanghai Pesticide Research Institute (Shanghai, China). Thiamethoxam (98.0% purity) and carbendazim (97.0% purity) standards were supplied by the Agro-Environmental Protection Institute of the Ministry of Agriculture and Rural Affairs (Tianjin, China). Detailed information about the physicochemical properties of the three pesticides is given in Table 1.

### 2.2. Instruments and Equipment

The following instruments were used in this study: LC-30AD liquid chromatograph and 8050 triple quadrupole mass spectrometer (Shimadzu Corporation, Kyoto, Japan); BSA2202S electronic balance (Sartorius AG, Goettingen, Germany); Biofuge Primo R benchtop centrifuge (Thermo Fisher Scientific, Waltham, MA, USA); VX-III multi-tube vortex oscillator (Beijing Tajin Technology Co., Ltd., Beijing, China); and Milli-Q ultrapure water system (Millipore Corporation, Boston, MA, USA).

### 2.3. Field Experiment Design and Sampling

The field trial was conducted in greenhouses at the experimental field of the Huzhou Academy of Agricultural Sciences (30°52′ N, 120°06′ E), Zhejiang Province, China, from August to December 2023. Cabbage seeds were sown on 28 August 2023, and transplanted on September 16, and pesticide applications were performed on November 2 based on the Guideline for the Testing of Pesticide Residues in Crops (NY/T 788-2018) issued by the Ministry of Agriculture and Rural Affairs of the People’s Republic of China [19]. The three pesticides—azoxystrobin (250 g/L suspension concentrate, Syngenta Nantong Crop Protection Co., Ltd., Nantong, Jiangsu, China), thiamethoxam (25% water-dispersible granules, Syngenta Crop Protection AG, Hangzhou, China), and carbendazim (50% wettable powder, Guangde, Sunong Biological Technology Co., Ltd., Guangde, Anhui, China)—were procured from local agricultural suppliers. Tank mixtures of these pesticides were applied using a knapsack sprayer at 2 dosage levels, as shown in Table 2. The experimental design was comprised of nine treatments, three replicates for recommended dosage level, three replicates for double dosage level, and three untreated control plots. Buffer zones were maintained between adjacent 50 m^2^ plots to prevent cross-contamination. Sampling was conducted at 2 h post application and 1, 3, 5, 7, 14, 21, and 28 d post application, with a minimum of 2 kg of samples collected per treatment group. It is worth noting that the application of pesticides at double dosage aligns with international guidelines (e.g., those from the Organisation for Economic Co-operation and Development (OECD) and the Joint FAO/WHO Meeting on Pesticide Residues (JMPR)) for residue trials. This approach evaluates worst-case scenarios, including potential misuse, spray drift, or uneven application, to determine maximum residue levels (MRLs) and assess dietary risks under exaggerated conditions. Crucially, it provides a conservative safety margin for risk assessment, ensuring recommended pre-harvest intervals (PHIs) remain protective even under irregular field practices. All trials were conducted under controlled conditions for scientific purposes only. All samples were labeled, processed, and stored at −20 °C for subsequent analysis.

### 2.4. Sample Preparation

A 10 g homogenized sample (accurately weighed to 0.01 g) was transferred to a 50 mL centrifuge tube. Then, 10 mL of acetonitrile was added, and the tubes were vortexed for 1 min. A quick, easy, cheap, effective, rugged, and safe (QuEChERS) extraction salt packet (containing 6 g anhydrous magnesium sulfate and 1.5 g sodium chloride) was added to the mixture. The tube was capped, subjected to manual vigorous shaking, and vortexed again for 1 min. After centrifugation at 4200 rpm for 5 min, 6 mL of the supernatant was transferred to a cleanup tube preloaded with 900 mg anhydrous magnesium sulfate, 150 mg PSA, and 150 mg C_18_ adsorbent. The mixture was vortexed for 1 min and centrifuged at 4200 rpm for 5 min. The resulting supernatant was filtered through a 0.22 μm microporous membrane. The filtrate was diluted 1:1 (*v*/*v*) with ultrapure water, vortexed for 10 s, and analyzed using liquid chromatography tandem mass spectrometry (LC-MS/MS). Based on the initial injection results, samples were appropriately diluted, and matrix-matched standard working solutions were prepared at corresponding dilution factors for quantitative calibration.

### 2.5. Integrated Chromatographic and Mass Spectrometric Conditions

Chromatographic separation was performed on a Luna C_18_ column (100 mm × 2.1 mm, 1.7-μm particle size) maintained at 35 °C using a mobile phase composed of (A) 5 mmol/L ammonium formate in ultrapure water and (B) methanol. A 10 min gradient elution program was applied as follows: 5% B to 40% B from 0.00 to 1.00 min, 40% B to 80% B from 1.00 to 3.00 min, 80% B to 95% B from 3.00 to 5.00 min, held at 95% B until 8.00 min, rapidly reduced to 5% B within 0.01 min, and re-equilibrated at 5% B for 1.99 min. The injection volume was 1 μL. An electrospray ionization source operated in positive ion mode with a voltage of 4000 V and an ion source temperature of 300 °C was used for mass spectrometric detection. Collision-induced dissociation utilized argon as the collision gas, and nitrogen served as both the nebulizing gas (3 L/min) and drying gas (10 L/min). Air was employed as the heating gas at a flow rate of 10 L/min. Data acquisition was conducted in multiple reaction monitoring mode to ensure selective ion tracking.

### 2.6. Method Validation

Standard solutions of the three pesticides were prepared at concentrations ranging from 0.01 to 10 mg/L and analyzed to validate linearity. Accuracy was determined as the average recovery using spiked blank samples. To this end, blank cabbage samples were spiked with the three pesticides at four different levels (0.01–150 mg/kg). Precision, expressed as the relative standard deviation (RSD), was determined from six replicate analyses at each spiking level. The limit of detection (LOD) was calculated based on a signal-to-noise ratio (S/N) of 3. The limit of quantification (LOQ) was defined as the lowest spiked level achieving satisfactory recovery (70–120%) and an RSD less than 20%. Matrix effects (ME) were evaluated from the slope ratios between the calibration curves obtained in matrix and in solvent: ME (%) = [(slope_matrix_/slope_solvent_) − 1] × 100%, where values within ±20% indicate acceptable matrix interference.

### 2.7. Theoretical Calculation

#### 2.7.1. Dissipation Kinetics

A first-order kinetic model was used to analyze the degradation dynamics and half-life (*t*_1/2_) of azoxystrobin, thiamethoxam, and carbendazim in cabbage. The following equations were used in their calculation:

(1) *C_t_* = *C*_0_*e*^−^*^kt^*

(2) *t*_1/2_ = (ln2)/*k*

where *C*_0_ (mg/kg) is the initial residue concentration; *C_t_* (mg/kg) is the residue concentration at time *t* (d) after the application of pesticides; and *k* is the degradation rate constant.

#### 2.7.2. Risk Assessment

The dietary exposure and risk assessment were calculated using the following equations [20]:

(3) *EED* = *CRL* × *FI/bw*

(4) *RQ* = *EED/ADI*

where *EED* is the estimated exposure dosage; *CRL* is the calculated residue level; *FI* is the food intake; RQ is the risk quotient; and *ADI* is the acceptable daily intake obtained from the National Food Safety Standard MRLs for Pesticides in Food [21]. The *ADI* values for azoxystrobin, thiamethoxam, and carbendazim are 0.2, 0.08, and 0.03 mg/kg body weight (bw), respectively, which serve as health guidance values for risk assessment.

An *RQ* < 1 indicates that the health risk is within an acceptable range, with lower *RQ* values corresponding to lower risks. Conversely, an *RQ* ≥ 1 suggests a potential risk to human health.

In the 2nd approach, the European Food Safety Authority (EFSA) Pesticide Residue Intake Model (EFSA PRIMo revision 3.1) was utilized to assess the chronic risk of pesticide residues in cabbage [22]. The model incorporates data from national food surveys conducted by Member States, including cabbage consumption data. To determine the chronic exposure as a percentage of the Acceptable Daily Intake (%ADI), the IEDI is divided by the ADI of the pesticides and multiplied by 100. If the resulting %ADI exceeds 100%, it indicates that the pesticide residues in cabbage may pose a risk to human health.

## 3. Results and Discussion

### 3.1. Method Performance and Quality Assurance

Standard solutions of azoxystrobin, thiamethoxam, and carbendazim were prepared in acetonitrile–water (1:1, *v*/*v*) to establish solvent-based calibration curves (concentration range of 0.01–10.0 mg/L for all analytes). Matrix-matched standard solutions were similarly prepared by dissolving the pesticides in extracts of the cabbage matrix, with identical concentration ranges as the solvent-based standards. Samples whose analyte concentrations exceeded the calibration range (0.01–10.0 mg/L) were appropriately diluted before quantification using the established calibration curves.

As shown in Table 3, the R^2^ values of the solvent calibration curves for azoxystrobin, thiamethoxam, and carbendazim were 0.9986, 0.9998, and 0.9996, respectively, and the R^2^ values of their calibration curves in the cabbage matrix were 0.9991, 0.9988, and 0.9991, respectively, all showing a good linear correlation. Azoxystrobin, thiamethoxam, and carbendazim exhibited negligible matrix effects in cabbage, showing values of −17.51, −12.95, and −14.18%, respectively. The recovery and relative standard deviation (RSD) of azoxystrobin, thiamethoxam, and carbendazim are shown in Table 4. In pesticide-free blank cabbage matrices, standard solutions of azoxystrobin (0.01, 0.1, 5, and 20 mg/kg), thiamethoxam (0.01, 0.1, 5, and 20 mg/kg), and carbendazim (0.01, 0.1, 10, and 150 mg/kg) were prepared, with six replicates per concentration level. Following extraction and purification, the average recoveries of the three pesticides ranged from 82.5 to 104.2%, with RSDs of 2.1–5.2%, which are suitable for pesticide residue analysis of azoxystrobin, thiamethoxam, and carbendazim residues in accordance with guidelines for the testing of pesticide residues in crops (NY/T 788-2018) [19]. The limit of detection (LOD) for the three target pesticides was determined as 0.002 mg/kg, based on the lowest concentration point of the standard curve and the sample pretreatment dilution factor. The limit of quantification (LOQ) was set to 0.01 mg/kg for azoxystrobin, thiamethoxam, and carbendazim, corresponding to the lowest spiked level. The developed method demonstrated satisfactory linearity, accuracy, and precision, complying with the performance criteria for pesticide residue analysis.

### 3.2. Dissipation of Three Pesticides in Cabbage

**Figure 1:**
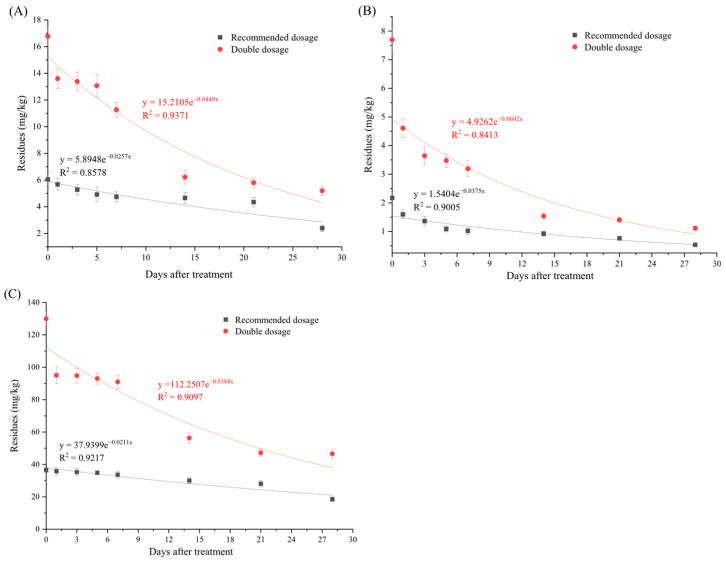
Residual dynamic curve of three pesticides in cabbage. (**A**–**C**) represent azoxystrobin, thiamethoxam, and carbendazim, respectively.

To develop an effective plant protection strategy, disease- and pest-control agents were simultaneously applied to cabbage plants. In this study, the pesticide concentrations were measured at various stages of cabbage growth (2 h, 1, 3, 5, 7, 14, 21, and 28 d post application). Table S1 and Figure 1 show the dissipation dynamics and parameters of the three pesticides in cabbage under greenhouse conditions. Variations in the initial deposition amounts were observed among the pesticides, with carbendazim showing the highest deposition, followed by azoxystrobin and thiamethoxam. The correlation coefficient (R^2^ = 0.841–0.937) for each fitting curve showed that the degradation kinetics of the three pesticides in cabbage followed first-order kinetics (Table 5). The initial azoxystrobin, thiamethoxam, and carbendazim concentrations at the recommended dosages were 6.06, 2.17, and 36.55 mg/kg, respectively, with corresponding half-lives of 26.65, 18.24, and 33.00 d, respectively. At double dosages, the initial concentrations were 16.77, 7.70, and 129.97 mg/kg, respectively, with corresponding half-lives of 15.40, 11.55, and 17.77 d, respectively. Overall, the original deposition amounts of all three pesticides increased significantly with increasing application dosages. As the sampling interval was extended, the residue levels progressively decreased relative to the initial deposition amount. At 28 d after treatment, azoxystrobin, thiamethoxam, and carbendazim exhibited dissipation rates of 60.50, 75.37, and 49.32%, respectively, under recommended dosages, and increased rates of 68.97, 85.52, and 64.16%, respectively, at double dosages.

In our study, the half-lives of azoxystrobin, thiamethoxam, and carbendazim in cabbage ranged from 15.40 to 33.00 d, which were significantly longer than those reported in other vegetables including scallions, cucumbers, tomatoes, curry leaves, and watercress (1.0–7.0 d) [23,24,25,26,27,28]. For example, Li et al. [29] reported that thiamethoxam degradation in Chinese kale exceeded 50% by 7 d post application, while Karmakar and Kulshrestha [30] found that degradation loss in the tomato reached 82–87% by 10 d post application, with a half-life of 4 d. This phenomenon is reasonable as the pesticide dissipation rates are closely related to crop variety and cultivation climate.

Consistent with previous studies, our data confirm that pesticide residue levels are highly sensitive to agronomic and environmental variables, particularly crop species and application weather conditions (temperature-driven degradation and wind-induced drift) [31,32]. Under environmentally homogenous conditions, pesticidal attenuation mechanisms become principally governed by compound-specific physicochemical profiles [33,34,35]. In our study, thiamethoxam demonstrated a shorter dissipation half-life than azoxystrobin and carbendazim did. This accelerated degradation was attributed to its lower Log K_ow_ (−0.13) and higher aqueous solubility (4.1 g/L), both of which are physicochemical properties that limit bioaccumulation potential and promote environmental decomposition [36].

Pesticide dissipation primarily occurs through two pathways: the first is volatilization, which refers to the direct evaporation of pesticides from plant surfaces; the second involves internal plant losses, encompassing both pesticide depletion due to transpiration (water loss through leaf stomata) and biotransformation processes [37,38]. Thiamethoxam, a highly polar pesticide with high water solubility, can migrate through the soil water phase to plant roots and subsequently be transported upward through the xylem via transpiration to stems and leaves [39].

Additionally, after absorbing xenobiotics, plants trigger detoxification defense mechanisms. Cytochrome P450 (CYP450), Carboxylesterase (CarE), and Glutathione S-transferase (GST) are metabolic enzymes in plants that play significant roles in the metabolism and absorption of substances [40,41]. Wang et al. [42] revealed that competitive inhibition of GST enzymes by thiamethoxam and bifenthrin during co-application diminished the degradation efficiency, prolonging the dissipation half-lives of these insecticides and altering their metabolic pathways. Similarly, pesticide degradation in plants may be influenced by enzymatic interactions, given their capacity to positively or negatively modulate plant enzyme activity. Previous research has shown that catalase expression enhances malathion degradation in cucumber [43]. Yu et al. [44] demonstrated that glutathione reduced chlorothalonil residues in tomato by enhancing antioxidative enzyme activity, increasing non-enzymatic substance levels, and activating detoxification gene expression. Thus, the longer *t*_1/2_ of the three target pesticides in cabbage may be attributed to enzymatic inhibition mechanisms. The longer *t*_1/2_ of pesticides in greenhouse vegetables may be attributed to specific climatic conditions, such as poor ventilation, insufficient light exposure, and excessive humidity, which can impede the photodegradation and evaporation processes of pesticides [45].

### 3.3. Terminal Residual Levels of Three Pesticides in Cabbage

The terminal residues of azoxystrobin, thiamethoxam, and carbendazim in cabbage sampled at different intervals (2 h, 1, 3, 5, 7, 14, 21, and 28 d) are summarized in Table S1 and Figure 1. When azoxystrobin, thiamethoxam, and carbendazim were applied at a lower dosage (recommended dosages), the terminal residues ranged from 2.39 to 5.68, 0.53 to 1.60, and 18.52 to 35.75 mg/kg, respectively; when applied at a high dosage (double dosages), the terminal residues ranged from 5.21 to 13.6, 1.12 to 4.61, and 46.58 to 95.08 mg/kg, respectively. The residues of the three pesticides in cabbage decreased with the increase in time after application, and at the same harvest time, the amount of residue at the high dosage was higher than that at the low dosage. Although residue levels gradually decreased with the extension of the pre-harvest interval (PHI), the decline was not significant, which may be attributed to the combined effects of the simultaneous application of three pesticides and their relatively longer half-lives under greenhouse conditions [46,47]. The three pesticides were applied during the maturity stage of cabbage when its metabolism had slowed, resulting in slower degradation rates. This view is also supported by the study of pesticide residue degradation in leafy vegetables by Qi et al. [48]. Qian et al. [49] demonstrated that when carbendazim was applied in combination with other pesticides, the residual concentration on peach at 35 d after spraying ranged from 4.26 to 7.50 mg/kg, with a half-life of 23.2–32.8 d. These findings are consistent with the results of our research.

As the primary site of pesticide application and the main organ for plant metabolism, leaves exhibit higher pesticide residues than other tissues [50]. A 15-year monitoring study conducted by Park et al. [11] in South Korea’s largest leafy vegetable production region revealed that 2.4% of the sampled leafy vegetables exceeded the maximum residue limit (MRL). Monitoring of vegetables sampled from local markets in Kuala Lumpur, Malaysia, revealed that 13.3% of samples exceeded the MRL, with carbendazim being the most frequently detected pesticide [51]. The MRLs set by China for specific pesticides in food are scientifically derived toxicological safety levels that reflect necessary and correct agricultural use under Good Agricultural Practice (GAP), align with international standards, and suit China’s agricultural realities. These limits serve as a critical instrument for balancing effective crop protection with the overriding priority of food safety. According to the national food safety standard of China (GB 2763–2021), the MRLs for azoxystrobin and thiamethoxam in cabbage are 5 and 0.2 mg/kg, respectively [21]. The azoxystrobin residue level (4.92 mg/kg) detected 5 d post application was below the Chinese MRL of 5 mg/kg; in contrast, the thiamethoxam residue level (0.53 mg/kg) measured 28 d post application exceeded the Chinese MRL of 0.2 mg/kg. An MRL has not been established for carbendazim in cabbage in China. The European Union (EU), Japan, and the United States (US) have set MRLs for cabbage of 5, 5, and 3 mg/kg, respectively, for azoxystrobin and 0.02, 3, and 4.5 mg/kg, respectively, for thiamethoxam; the EU and Japan have set MRLs of 0.1 and 3 mg/kg, respectively, for carbendazim [52,53]. At 28 d post application, carbendazim residues in cabbage were significantly higher than the MRLs set by the EU and Japan, regardless of whether the recommended or double dosage was used. Based on this, we express concern about the potential food safety risks associated with the use of carbendazim in cabbage cultivation.

### 3.4. Health Risk Assessment of Pesticide Residues in Cabbage

Thiamethoxam is a globally widely used insecticide that has been registered in China for protecting cabbage against aphids, whiteflies, and striped flea beetles. In China, although azoxystrobin and carbendazim are not currently registered for use on cabbage, carbendazim is approved for controlling Sclerotinia stem rot in rapeseed, and azoxystrobin can be applied for anthracnose in radish and downy mildew in cauliflower, among other cruciferous vegetable diseases. To evaluate the health risks posed by three target pesticides (azoxystrobin, thiamethoxam, and carbendazim), a health risk assessment for cabbage ingestion was conducted. The assessment quantified consumer exposure risk using RQ based on the mean residue concentrations of these pesticides in cabbage samples. The assessment considered the following: (1) differences in bw between children (32.7 kg) and adults (55.9 kg) [54]; and (2) the principle of maximum risk, in which cabbage intake was based on the recommended daily vegetable consumption (0.1085 kg for children and 0.242 kg for adults) [55]. The dietary risk probability was assessed using RQ, which was calculated by comparing the value of estimated exposure dosage (EED) of azoxystrobin, thiamoxam, and carbendazim with acceptable daily intake (ADI), based on the terminal residual data from field experiments. As shown in Table 6, the RQs of the three pesticides showed a decreasing trend over time.

Table 6 shows the RQ of pesticide residues in cabbage at different harvest intervals for both the recommended and double dosage applications. The experimental results for both children and adults showed the following: (1) at 1 d after azoxystrobin application, the RQ values were 0.094 (children) and 0.123 (adults) at the recommended dosage and 0.226 (children) and 0.294 (adults) at the double dosage, indicating acceptable health risks, as all RQ values were below 1; (2) at 1 d after thiamethoxam application, the RQ values were 0.066 (children) and 0.086 (adults) at the recommended dosage and 0.191 (children) and 0.249 (adults) at the double dosage, demonstrating acceptable health risks; (3) at 28 d after carbendazim application, the RQ values reached 2.049 (children) and 2.673 (adults) at the recommended dosage and increased to 5.152 (children) and 6.722 (adults) at the double dosage, indicating unacceptable health risks, with all values exceeding 1. The results indicate that azoxystrobin and thiamethoxam residues in cabbage pose no health risks to adults or children. However, for cabbage treated with carbendazim at both dosage levels, the RQs revealed a high dietary risk across all studied age groups.

In the second approach (EFSA PRIMo version 3.1), the concentrations of the three pesticides at recommended and double dosages on the 28th day were used for dietary risk assessment. The ADIs of azoxystrobin, thiamethoxam, and carbendazim were set at 0.2, 0.026, and 0.02 mg/kg bw/day, respectively [52]. The chronic risk assessment for populations of different nationalities and age groups consuming cabbage reveals several concerning findings. The %ADI values for azoxystrobin at recommended and double dosages are in the range of 0.01–1.71% and 0.01–3.73%, respectively. The %ADI values for thiamethoxam at recommended and double dosages are in the range of 0.01–2.92% and 0.02–6.17%, respectively. The %ADI values for carbendazim at recommended and double dosages are in the range of 0.47–132.73% and 1.18–333.82%, respectively. As shown in Table 7, azoxystrobin and thiamethoxam demonstrate very low exposure levels, with the estimated intakes of azoxystrobin and thiamethoxam generally below 10% of the ADI and even lower percentages of the ADI at the recommended dosage. In contrast, carbendazim exhibits substantially higher %ADI values. Specifically, under the recommended dosage, the RO general yields a %ADI value as high as 132.73%. At double dosage, the %ADI values for population groups (RO general, SE general, GEMS/Food G08, and GEMS/Food G15) are 333.82%, 145.56%, 105.27%, and 190.82%, respectively. This indicates that at current residue levels, chronic dietary exposure risk from carbendazim intake via cabbage is high, posing serious health risks to consumers. These findings align with our risk assessment results using the RQ.

Carbendazim, a fungicide with both protective and therapeutic effects, is widely used to control fungal diseases in vegetables, fruit, tea, and other crops. It is a pesticide with a high residue detection rate in agricultural products [56,57]. Wang et al. [14] investigated carbendazim residues in plant-based foods in China from 2011 to 2020. The survey revealed that residues exceeding 10 mg/kg were detected in 10 vegetable varieties; among these, leafy vegetables exhibited the highest detection rate (16–25%), with maximum concentrations reaching 110 mg/kg, which is consistent with our results. The surface-area-to-mass ratio (specific surface area) of leafy vegetables, which was higher than that of other vegetable types (solanaceous vegetables, cucurbit vegetables, and bulb vegetables), significantly enhances pesticide retention by expanding the area of adsorbing interfaces [58].

Carbendazim should not be rotated with fungicides of the same class (e.g., thiophanate, benomyl, and methyl thiophanate), as prolonged exclusive use or rotation with such analogs may lead to pathogen resistance. Given its slow degradation rate in greenhouse-grown cabbage and the high detection rates in other fruit and vegetable crops—factors that may contribute to human accumulation—the use of carbendazim in cabbage cultivation is not recommended to avoid the risks associated with multi-pesticide residue coexistence and combined toxicity.

Although the individual residue risks of azoxystrobin and thiamethoxam in cabbage are acceptable (RQ < 1), the high carbendazim residue levels may significantly amplify combined exposure risks. Given carbendazim’s persistent residues (*t*_1/2_ > 30 d), which may lead to prolong combined exposure with other pesticides, and considering that pesticides with distinct modes of action can have synergistic effects through metabolic interference or toxicity superposition (e.g., hepatotoxicity or neurotoxicity), the monitoring and assessment of multi-pesticide residue synergism should be strengthened to comprehensively ensure agricultural product safety.

It should be noted that all field trials were conducted in single-span plastic greenhouses in East China during the autumn season and examined only the dissipation kinetics and dietary risks associated with the specific combination of azoxystrobin, thiamethoxam, and carbendazim applied to cabbage. Because greenhouse type (multi-span vs. single-span), temperature and humidity management, light intensity, and soil properties can markedly influence pesticide degradation dynamics, extrapolation of these results to different climatic regions, greenhouse structures, or other Brassicaceae crops should be undertaken with caution. Moreover, although the simultaneous application of the three pesticides mimics some agricultural practices, the study did not encompass all possible dose ratios or application frequencies, and therefore, the findings cannot be directly extended to alternative combinations or scenarios.

## 4. Conclusions

This study systematically evaluated the dissipation dynamics, final residue levels, and dietary intake risks of azoxystrobin, thiamethoxam, and carbendazim in cabbage under greenhouse cultivation conditions, following their combined application at recommended and double dosages. The dissipation of residues followed first-order kinetics with half-lives of 11.55–33.00 d for the selected pesticides. The residue concentrations of three pesticides in cabbage decreased with the extension of the PHI in the different trials, and at the same harvest time, the residues after double dosage application were higher than those after recommended dosage application. The dietary intake risks of azoxystrobin and thiamethoxam (recommended and double dosages) were acceptable for children and adults. In cabbage, the residual carbendazim concentrations remained greater than 10 mg/kg at 28 d post application under both recommended and double dosage treatments, significantly exceeding the MRLs set by the EU (0.01 mg/kg) and Japan (3 mg/kg). Furthermore, dietary risk assessments revealed unacceptable exposure levels, with RQ > 1 and %ADI exceeding 100%, indicating potential health risks. The combined application of these pesticides may pose heightened cumulative risks through potential synergistic interactions and prolonged co-exposure, as carbendazim’s persistent residues could exacerbate cumulative toxicity when occurring together with azoxystrobin and thiamethoxam over extended periods; however, this remains a potential outcome pending direct synergism testing.

Given the high detection frequency of carbendazim in fruit and vegetable crops and its prolonged degradation half-life in cabbage (*t*_1/2_ = 33 d under the recommended dosage), carbendazim use should be restricted in cabbage cultivation. This recommendation is based on the following: (1) toxicological evidence: carbendazim is classified as an EU CMR 1B carcinogen with endocrine-disrupting properties [59]; (2) regulatory precedents: it is banned in the EU, UK, Brazil, and partially restricted in China for high-risk crops; (3) available alternatives: biopesticides (e.g., *Bacillus subtilis*) effectively control cabbage diseases without leaving persistent residues. The implementation of such restrictions would effectively mitigate cumulative health risks in greenhouse cabbage production systems.

## Figures and Tables

**Table 1:** Selected physicochemical properties of pesticides used in this study.

Pesticide	CAS Registry Number	Molecular Structure	Molecular Weight	Solubility (mg/L)	Log K_ow_
Azoxystrobin	131860-33-8	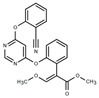	403.39	6	2.50
Thiamethoxam	153719-23-4	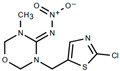	291.71	4100	−0.13
Carbendazim	10605-21-7	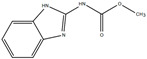	191.19	8	1.4–1.5

**Table 2:** Application dosages of three pesticides at two concentrations.

Pesticide	Recommended Dosage (g a.i./ha)	Double Dosage (g a.i./ha)
Azoxystrobin	150	300
Thiamethoxam	56.25	112.5
Carbendazim	750	1500

**Table 3:** Standard curves of the solvent and matrix of three pesticides.

Pesticide	Range of Linearity (mg/L)	Solvent Standard Curve	Matrix Standard Curve	Matrix Effect (%)
Azoxystrobin	0.01–10	*y* = 3.37 × 10^8^*x* + 3.86 × 10^5^ *R*^2^ = 0.9986	*y* = 2.78 × 10^8^*x* + 9.52 × 10^6^ *R*^2^ = 0.9991	−17.51
Thiamethoxam	0.01–10	*y* = 4.48 × 10^7^*x* + 6.88 × 10^4^ *R*^2^ = 0.9998	*y* = 3.90 × 10^8^*x* + 3.32 × 10^4^ *R*^2^ = 0.9988	−12.95
Carbendazim	0.01–10	*y* = 4.23 × 10^8^*x* + 2.94 × 10^5^ *R*^2^ = 0.9996	*y* = 3.63 × 10^8^*x* + 2.23 × 10^5^ *R*^2^ = 0.9991	−14.18

**Table 4:** Average recovery rate and relative standard deviation of three pesticides (n = 6).

Pesticide	Spink Concentration (mg/kg)	Average Recovery (%)	Relative Standard Deviation (%)
Azoxystrobin	0.01	93.04	3.11
	0.1	86.31	4.23
	5	102.41	4.52
	20	84.11	2.54
Thiamethoxam	0.01	104.23	3.56
	0.1	88.27	5.23
	5	93.55	4.15
	20	95.12	3.77
Carbendazim	0.01	88.33	4.23
	0.1	82.47	2.14
	10	88.29	3.53
	150	93.11	4.67

**Table 5:** Dissipation rate of the three pesticides in cabbage.

Pesticide	Treatment	Dynamic Equation	Correlation Coefficient (R^2^)	Half-Life (*t*_1/2_, days)
Azoxystrobin	Recommended dosage	C_t_ = 5.895e^−0.026x^	0.858	26.65
	Double dosage	C_t_ = 15.211e^−0.045x^	0.937	15.40
Thiamethoxam	Recommended dosage	C_t_ = 1.540e^−0.038x^	0.901	18.24
	Double dosage	C_t_ = 4.926e^−0.060x^	0.841	11.55
Carbendazim	Recommended dosage	C_t_ = 37.940e^−0.021x^	0.922	33.00
	Double dosage	C_t_ = 112.251e^−0.039x^	0.910	17.77

**Table 6:** Estimated exposure dosage and risk quotient of the three pesticides in cabbage.

Time After Spray (Days)	Individuals	Body Weight (kg)	Food Intake (kg/d)	EED (mg/kg bw)						RQ					
				Azoxystrobin		Thethiamoxam		Carbendazim		Azoxystrobin		Thethiamoxam		Carbendazim	
				Recommended Dosage	Double Dosage	Recommended Dosage	Double Dosage	Recommended Dosage	Double Dosage	Recommended Dosage	Double Dosage	Recommended Dosage	Double Dosage	Recommended Dosage	Double Dosage
2 h	Children	32.7	0.1085	0.020	0.056	0.007	0.026	0.121	0.431	0.100	0.278	0.090	0.319	4.043	14.375
	Adults	55.9	0.242	0.026	0.073	0.009	0.033	0.158	0.563	0.131	0.363	0.117	0.417	5.274	18.755
1	Children	32.7	0.1085	0.019	0.045	0.005	0.015	0.119	0.315	0.094	0.226	0.066	0.191	3.954	10.516
	Adults	55.9	0.242	0.025	0.059	0.007	0.020	0.155	0.412	0.123	0.294	0.086	0.249	5.159	13.720
3	Children	32.7	0.1085	0.018	0.044	0.005	0.012	0.117	0.315	0.088	0.222	0.057	0.151	3.903	10.487
	Adults	55.9	0.242	0.023	0.058	0.006	0.016	0.153	0.410	0.114	0.29	0.074	0.197	5.092	13.683
5	Children	32.7	0.1085	0.016	0.043	0.004	0.012	0.115	0.309	0.082	0.217	0.045	0.144	3.849	10.291
	Adults	55.9	0.242	0.021	0.057	0.005	0.015	0.151	0.403	0.106	0.283	0.059	0.188	5.023	13.427
7	Children	32.7	0.1085	0.016	0.037	0.003	0.011	0.112	0.302	0.079	0.187	0.042	0.132	3.724	10.060
	Adults	55.9	0.242	0.021	0.049	0.004	0.014	0.146	0.394	0.103	0.244	0.055	0.173	4.859	13.126
14	Children	32.7	0.1085	0.015	0.021	0.003	0.005	0.100	0.187	0.077	0.103	0.038	0.064	3.322	6.228
	Adults	55.9	0.242	0.020	0.027	0.004	0.007	0.130	0.244	0.101	0.135	0.050	0.083	4.334	8.126
21	Children	32.7	0.1085	0.014	0.019	0.003	0.005	0.093	0.157	0.072	0.096	0.032	0.058	3.108	5.222
	Adults	55.9	0.242	0.019	0.025	0.003	0.006	0.122	0.204	0.094	0.126	0.041	0.076	4.056	6.813
28	Children	32.7	0.1085	0.008	0.017	0.002	0.004	0.061	0.155	0.040	0.086	0.022	0.046	2.049	5.152
	Adults	55.9	0.242	0.010	0.023	0.002	0.005	0.080	0.202	0.052	0.113	0.029	0.060	2.673	6.722

**Table 7:** Chronic risk assessment for populations of different nationalities and age groups consuming pesticide-contaminated cabbage: IEDI (%ADI) according to the European Food Safety Authority Pesticide Residue Intake Model version 3.1 (EFSA PRIMo 3.1).

Population Group	Consumption (g/kg bw/d)	Body Weight (kg)	Azoxystrobin (%ADI)		Thiamethoxam (%ADI)		Carbendazim (%ADI)	
			Recommended Dosage	Double Dosage	Recommended Dosage	Double Dosage	Recommended Dosage	Double Dosage
DE child	0.09	16.2	0.11%	0.23%	0.18%	0.39%	8.33%	20.96%
DK child	0.07	21.8	0.09%	0.19%	0.15%	0.31%	6.73%	16.94%
ES child	0.05	34.5	0.06%	0.13%	0.10%	0.21%	4.56%	11.46%
FR toddler 2 3 yr	0.01	13.6	0.01%	0.01%	0.01%	0.02%	0.47%	1.18%
FR child 3 15 yr	0.04	18.9	0.05%	0.11%	0.09%	0.19%	3.98%	10.02%
IT toddler	0.01	41.6	0.01%	0.01%	0.01%	0.02%	0.47%	1.18%
NL toddler	0.15	10.2	0.17%	0.38%	0.30%	0.62%	13.43%	33.77%
NL child	0.14	18.4	0.17%	0.38%	0.29%	0.62%	13.33%	33.54%
UK infant	0.10	8.7	0.12%	0.27%	0.21%	0.45%	9.58%	24.09%
UK toddler	0.10	14.6	0.11%	0.25%	0.20%	0.41%	8.88%	22.33%
DK adult	0.08	75.1	0.10%	0.22%	0.17%	0.36%	7.75%	19.49%
ES adult	0.03	68.5	0.04%	0.08%	0.07%	0.14%	2.98%	7.50%
FI adult	0.10	78.1	0.12%	0.27%	0.21%	0.44%	9.53%	23.96%
FR adult	0.03	66.4	0.04%	0.08%	0.06%	0.14%	2.94%	7.39%
IE adult	0.14	75.2	0.16%	0.36%	0.28%	0.59%	12.68%	31.90%
IT adult	0.03	66.5	0.03%	0.07%	0.05%	0.11%	2.39%	6.02%
LT adult	0.40	70.0	0.48%	1.04%	0.81%	1.72%	36.91%	92.83%
NL general	0.18	65.8	0.21%	0.47%	0.36%	0.77%	16.58%	41.69%
PL general	0.36	62.8	0.44%	0.95%	0.74%	1.57%	33.79%	84.99%
RO general	1.43	60.0	1.71%	3.73%	2.92%	6.17%	132.73%	333.82%
SE general	0.63	60.0	0.75%	1.63%	1.27%	2.69%	57.88%	145.56%
UK adult	0.07	76.0	0.09%	0.19%	0.15%	0.31%	6.70%	16.85%
UK vegetarian	0.10	66.7	0.12%	0.27%	0.21%	0.44%	9.44%	23.74%
GEMS/Food G06	0.17	60.0	0.20%	0.45%	0.35%	0.74%	15.82%	39.79%
GEMS/Food G07	0.15	60.0	0.18%	0.39%	0.30%	0.64%	13.84%	34.82%
GEMS/Food G08	0.45	60.0	0.54%	1.18%	0.92%	1.95%	41.86%	105.27%
GEMS/Food G10	0.42	60.0	0.50%	1.08%	0.85%	1.79%	38.52%	96.89%
GEMS/Food G11	0.08	60.0	0.09%	0.20%	0.15%	0.33%	7.02%	17.66%
GEMS/Food G15	0.82	60.0	0.98%	2.13%	1.67%	3.53%	75.87%	190.82%
DE general	0.16	76.4	0.19%	0.42%	0.33%	0.70%	14.95%	37.60%
DE women 14–50 yr	0.13	67.5	0.15%	0.33%	0.26%	0.55%	11.86%	29.82%
IE child	0.03	20.0	0.03%	0.07%	0.05%	0.11%	2.39%	6.02%
FI 3 yr	0.07	15.2	0.09%	0.20%	0.15%	0.32%	6.94%	17.46%
FI 6 yr	0.08	22.4	0.10%	0.21%	0.17%	0.35%	7.59%	19.08%

Notes: IEDI—International Estimated Daily Intake; ADI—acceptable daily intake; DE—Germany; DK—Denmark; ES—Spain; FR—France; IT–Italy; NL—Netherlands; UK—United Kingdom; FI—Finland; IE—Ireland; IT—Italy; LT—Lithuania; PL—Poland; RO—Romania; SE—Sweden; GEMS/Food G06: general population (cluster diet 06 covers Greece); GEMS/Food G07: general population (cluster diet 07 covers Finland, France, Luxembourg, and the United Kingdom); GEMS/Food G08: general population (cluster diet 08 covers Austria, Germany, Poland, and Spain); GEMS/Food G10: general population (cluster diet 10 covers Bulgaria, Croatia, Cyprus, Estonia, Italy, Latvia, and Malta); GEMS/Food G11: general population (cluster diet 11 covers Belgium and the Netherlands); GEMS/Food G15: general population (cluster diet 15 covers the Czech Republic, Denmark, Hungary, Ireland, Lithuania, Portugal, Romania, Slovakia, Slovenia, and Sweden); yr—years.

## Data Availability

The original contributions presented in the study are included in the article/Supplementary Material, further inquiries can be directed to the corresponding author.

## Supplementary Materials

The following supporting information can be downloaded at https://www.mdpi.com/article/10.3390/foods14173006/s1, Table S1: Residues of three pesticides in cabbage.

## References

[1] Li C.J., Zhu H.M., Li C.Y., Qian H., Yao W.R., Guo Y.H. (2021). The present situation of pesticide residues in China and their removal and transformation during food processing. Food Chem..

[2] FAO (2025). FAOSTAT.

[3] Fradejas-García I., Molina J.L., Lubbers M.J. (2024). Migrant entrepreneurs in the ‘Farm of Europe’: The role of transnational structures. Globalizations.

[4] Zhang Z.L., Sun D., Tang Y., Zhu R., Li X., Gruda N., Dong J.L., Duan Z.Q. (2021). Plastic shed soil salinity in China: Current status and next steps. J. Clean. Prod..

[5] Franzke A., Lysak M.A., Al-Shehbaz I.A., Koch M.A., Mummenhoff K. (2011). Cabbage family affairs: The evolutionary history of Brassicaceae. Trends Plant Sci..

[6] Zhu M.Z., Wang Y., Lu S.J., Yang L.M., Zhuang M., Zhang Y.Y., Lv H.H., Fang Z.Y., Hou X.L. (2022). Genome-wide identification and analysis of cytokinin dehydrogenase/oxidase (CKX) family genes in Brassica oleracea L. reveals their involvement in response to Plasmodiophora brassicae infections. Hortic. Plant J..

[7] Verkerk R., Dekker M., Jongen W.M.F. (2001). Post-harvest increase of indolyl glucosinolates in response to chopping and storage of Brassica vegetables. J. Sci. Food Agric..

[8] Guerrero-Beltrán C.E., Calderón-Oliver M., Pedraza-Chaverri J., Chirino Y.I. (2012). Protective effect of sulforaphane against oxidative stress: Recent advances. Exp. Toxicol. Pathol..

[9] Herr I., Buchler M.W. (2010). Dietary constituents of broccoli and other cruciferous vegetables: Implications for prevention and therapy of cancer. Cancer Treat. Rev..

[10] Wood P.M., Hollomon D.W. (2003). A Critical Evaluation of the Role of Alternative Oxidase in the Performance of Strobilurin and Related Fungicides Acting at Qo Site of Complex III. Pest Manag. Sci..

[11] Park D.W., Yang Y.S., Lee Y.U., Han S.J., Kim H.J., Kim S.H., Kim J.P., Cho S.J., Lee D., Song N. (2021). Pesticide Residues and Risk Assessment from Monitoring Programs in the Largest Production Area of Leafy Vegetables in South Korea: A 15-Year Study. Foods.

[12] Maienfisch P., Angst M., Brandl F., Fischer W., Hofer D., Kayser H., Kobel W., Rindlisbacher A., Senn R., Steinemann A. (2001). Chemistry and biology of thiamethoxam: A second generation neonicotinoid. Pest Manag. Sci..

[13] Zhou Y.J., Xu J.Q., Zhu Y.Y., Duan Y.B., Zhou M.G. (2016). Mechanism of action of the benzimidazole fungicide on fusarium graminearum: Interfering with polymerization of monomeric tubulin but not polymerized microtubule. Phytopathology.

[14] Wang D., Yang G.L., Yun X., Luo T., Guo H., Pan L.Y., Du W., Wang Y.H., Wang Q., Wang P. (2024). Carbendazim residue in plant-based foods in China: Consecutive surveys from 2011 to 2020. Environ. Sci. Ecotechnol..

[15] Zhang Y.K., Wang H., Wang X., Hu B., Zhang C.F., Jin W., Zhu S.J., Hu G., Hong Q. (2017). Identification of the key amino acid sites of the carbendazim hydrolase (MheI) from a novel carbendazim-degrading strain Mycobacterium sp SD-4. J. Hazard. Mater..

[16] Singh S., Singh N., Kumar V., Datta S., Wani A.B., Singh D., Singh K., Singh J. (2016). Toxicity, monitoring and biodegradation of the fungicide carbendazim. Environ. Chem. Lett..

[17] Fang H., Wang Y.Q., Gao C.M., Yan H., Dong B., Yu Y.L. (2010). Isolation and characterization of Pseudomonas sp CBW capable of degrading carbendazim. Biodegradation.

[18] (2020). Ministry of Agriculture and Rural Affairs of the People’s Republic of China, Green Food—Guideline for Application of Pesticide.

[19] (2018). Ministry of Agriculture and Rural Affairs of the People’s Republic of China, Guideline for the Testing of Pesticide Residues in Crops.

[20] Liu C.Y., Lu D.H., Wang Y.C., Huang J.X., Wan K., Wang F.H. (2014). Residue and risk assessment of pyridaben in cabbage. Food Chem..

[21] (2021). National Food Safety Standard-Maximum Residue Limits for Pesticides in Food; Ministry of Agriculture and Rural Affairs of the People’s Republic of China.

[22] Anastassiadou M., Brancato A., Carrasco Cabrera L., Ferreira L., Greco L., Jarrah S., Kazocina A., Leuschner R., Magrans J.O., European Food Safety Authority (2019). Pesticide Residue Intake Model-EFSA PRIMo Revision 3.1.

[23] Chai Y.D., Liu R., Du X.Y., Yuan L.F. (2022). Dissipation and Residue of Metalaxyl-M and Azoxystrobin in Scallions and Cumulative Risk Assessment of Dietary Exposure to Hepatotoxicity. Molecules.

[24] Elhefny D.E., Monir H.H., Helmy R.M.A. (2021). Validation using QuEChERS method, risk assessment and preharvest intermission using GC-MS for determination of azoxystrobin in tomato and cucumber. Egypt. J. Chem..

[25] Kaithamalai B., Palanisamy K., Chellamuthu S., Pandi T., Samygounder I., Venkidusamy M. (2024). Dissipation kinetics and decontamination of chlorantraniliprole and thiamethoxam residues in curry leave. Int. J. Environ. Anal. Chem..

[26] Al Dhafar Z.M., Abdel Razik M.A.A., Osman M.A., Sweelam M.E. (2023). Determination of thiamethoxam residues and dissipation kinetic in tomato plants and its efficacy against Bemisia tabaci under open field eco system. Braz. J. Biol..

[27] Dong B.Z., Hu J.Y. (2023). Residue dissipation and dietary intake risk assessment of thiophanate-methyl and its metabolite carbendazim in watercress under Chinese field conditions. Int. J. Environ. Anal. Chem..

[28] Liu Z., Chen Y., Han J.H., Chen D., Yang G.Q., Lan T.T., Li J.M., Zhang K.K. (2021). Determination, dissipation dynamics, terminal residues and dietary risk assessment of thiophanate-methyl and its metabolite carbendazim in cowpeas collected from different locations in China under field conditions. J. Sci. Food Agric..

[29] Li K.L., Chen W.Y., Zhang M., Luo X.W., Liu Y., Zhang D.Y., Chen A. (2022). Monitoring residue levels and dietary risk assessment of thiamethoxam and its metabolite clothianidin for Chinese consumption of Chinese kale. J. Sci. Food Agric..

[30] Karmakar R., Kulshrestha G. (2009). Persistence, metabolism and safety evaluation of thiamethoxam in tomato crop. Pest Manag. Sci..

[31] Abdallah O.I., Abd El-Hamid R.M., Raheem E.H.A. (2019). Clothianidin residues in green bean, pepper and watermelon crops and dietary exposure evaluation based on dispersive liquid-liquid microextraction and LC–MS/MS. J. Consum. Prot. Food Saf..

[32] Besil N., Pérez-Parada A., Bologna F., Cesio M.V., Rivas F., Heinzen H. (2019). Dissipation of selected insecticides and fungicides applied during pre-harvest on mandarin and orange trees in Uruguay. Sci. Hortic..

[33] Oliva J., Cermeño S., Cámara M.A., Martínez G., Barba A. (2017). Disappearance of six pesticides in fresh and processed zucchini, bioavailability and health risk assessment. Food Chem..

[34] Saber A.N., Malhat F., Anagnostopoulos C., Kasiotis K.M. (2020). Evaluation of dissipation, unit-unit-variability and terminal residue of etoxazole residues in strawberries from two different parts in Egypt. J. Consum. Prot. Food Saf..

[35] Fu D.H., Zhang S.Y., Wang M., Liang X.Y., Xie Y.L., Zhang Y., Zhang C.H. (2020). Dissipation behavior, residue distribution and dietary risk assessment of cyromazine, acetamiprid and their mixture in cowpea and cowpea field soil. J. Sci. Food Agric..

[36] Tian F.J., Qiao C.K., Wang C.X., Pang T., Guo L.L., Li J., Pang R.L., Xie H.Z. (2022). The fate of thiamethoxam and its main metabolite clothianidin in peaches and the wine-making process. Food Chem..

[37] Li Z., Fantke P. (2023). Considering degradation kinetics of pesticides in plant uptake models: Proof of concept for potato. Pest Manag. Sci..

[38] Wang Y.H., Liu P.P., Yang G.L., Shu F., Chen C. (2024). Exploring the dynamic behaviors of five pesticides in lettuce: Implications for consumer health through field and modeling experiments. Food Chem..

[39] Feng X.X., Pan L.X., Xu T.H., Jing J., Zhang H.Y. (2019). Dynamic modeling of famoxadone and oxathiapiprolin residue on cucumber and Chinese cabbage based on tomato and lettuce archetypes. J. Hazard. Mater..

[40] Hillebrands L., Lamshoeft M., Lagojda A., Stork A., Kayser O. (2020). Evaluation of Callus Cultures to Elucidate the Metabolism of Tebuconazole, Flurtamone, Fenhexamid, and Metalaxyl-M in *Brassica napus* L. *Glycine max* (L.) Merr. *Zea mays* L. and *Triticum aestivum* L.. J. Agric. Food Chem..

[41] Xie J.Q., Tang W., Zhaob L., Liu S.R., Liu K., Liu W.P. (2019). Enantioselectivity and allelopathy both have effects on the inhibition of napropamide on Echinochloa crus-galli. Sci. Total Environ..

[42] Wang T.C., Qian Y.Z., Wang J.Q., Yin X.Y., Liang Q.F., Liao G.Q., Li X.B., Qiu J., Xu Y.Y. (2024). Comparison of combined dissipation behaviors and dietary risk assessments of thiamethoxam, bifenthrin, dinotefuran, and their mixtures in tea. Foods.

[43] Bian Y.L., Liu F.M., Chen F., Sun P. (2018). Storage stability of three organophosphorus pesticides on cucumber samples for analysis. Food Chem..

[44] Yu G.B., Chen Q.S., Chen F.Q., Liu H.L., Lin J.X., Chen R.A., Ren C.Y., Wei J.P., Zhang Y.X., Yang F.J. (2022). Glutathione promotes degradation and metabolism of residual fungicides by inducing UDP-Glycosyltransferase genes in tomato. Front. Plant Sci..

[45] Tang H.X., Ma L., Huang J.Q., Li Y.B., Liu Z.H., Meng D.Y., Wen G.Y., Dong M.F., Wang W.M., Zhao L. (2021). Residue behavior and dietary risk assessment of six pesticides in pak choi using QuEChERS method coupled with UPLC-MS/MS. Ecotoxicol. Environ. Saf..

[46] Zhu X.D., Jia C.H., Duan L.F., Zhang W., Yu P.Z., He M., Chen L., Zhao E.C. (2016). Residue behavior and dietary intake risk assessment of three fungicides in tomatoes (Lycopersicon esculentum Mill.) under greenhouse conditions. Regul. Toxicol. Pharm..

[47] Di S.S., Wang Y.H., Xu H., Wang X.Q., Yang G.L., Chen C., Yang X., Qian Y.Z. (2021). Comparison the dissipation behaviors and exposure risk of carbendazim and procymidone in greenhouse strawberries under different application method: Individual and joint applications. Food Chem..

[48] Qi F., Liu X., Deng Z.S., Lu Y.Y., Chen Y.J., Geng H., Zhang Q.C., Rao Q.X., Song W.G. (2023). Effects of Thiamethoxam and Fenvalerate Residue Levels on Light-Stable Isotopes of Leafy Vegetables. Foods.

[49] Qian M.R., Zhou M., Li Y., Wang D., Yao L.P., Wu H.Z., Yang G.L. (2023). The Dissipation Behavior and Risk Assessment of Carbendazim Under Individual and Joint Applications on Peach (*Amygdalus persica* L.). J. Food Prot..

[50] Bauer A., Luetjohann J., Hanschen F.S., Schreiner M., Kuballa J., Jantzen E., Rohn S. (2018). Identification and characterization of pesticide metabolites in Brassica species by liquid chromatography travelling wave ion mobility quadrupole time-of-flight mass spectrometry (UPLC-TWIMS-QTOF-MS). Food Chem..

[51] Ishak A., Pak-Dek M.S., Rukayadi Y., Ramli N.S., Wasoh H. (2023). Evaluation of pesticide residues in selected vegetables from Kuala Lumpur, Malaysia using modified QuEChERS and assessment of washing methods. Int. Food Res. J..

[52] EU (2025). EU Pesticides Database. https://ec.europa.eu/food/plant/pesticides/eu-pesticides-database/mrls/?event=search.pr.

[53] JFCRF (2021). Maximum Residue Limits (MRLs) List of Agricultural Chemicals in Foods.

[54] Zheng N., Wang Q.C., Zhang X.W., Zheng D.M., Zhang Z.S., Zhang S.Q. (2007). Population health risk due to dietary intake of heavy metals in the industrial area of Huludao city, China. Sci. Total Environ..

[55] Li H., Chang Q.Y., Bai R.B., Lv X.C., Cao T.L., Shen S.G., Liang S.X., Pang G.F. (2021). Simultaneous determination and risk assessment of highly toxic pesticides in the market-sold vegetables and fruits in China: A 4-year investigational study. Ecotoxicol. Environ. Saf..

[56] Liu Y.H., Bei K., Zheng W.R., Yu G.G., Sun C.X. (2023). Pesticide residues risk assessment and quality evaluation of four characteristic fruits in Zhejiang Province, China. Front. Environ. Sci..

[57] Fan J.C., An J., Ren R., Liu S.Y., He H.L., Zhao G. (2023). Occurrence and exposure risk assessment of pesticide residues in green tea samples cultivated in Hangzhou area, China. Food Addit. Contam. Part B.

[58] Farha W., El-Aty A.M.A., Rahman M.M., Jeong J.H., Shin H., Wang J., Shin S.S., Shim J.H. (2018). Analytical approach, dissipation pattern, and risk assessment of pesticide residue in green leafy vegetables: A comprehensive review. Biomed. Chromatogr..

[59] Lu Z.J., Shi W.J., Qiao L.K., Ma D.D., Zhang J.G., Yao C.R., Li S.Y., Long X.B., Ying G.G. (2025). Benzimidazole Fungicide Carbendazim Induces Gut Inflammation through the TLR5/NF-κB Pathway in Grass Carp. Environ. Sci. Technol..

