# Supplementary material for: Dissipation Behavior and Risk Assessment of Three Pesticide Residues Under Combined Application in Greenhouse-Grown Cabbage
Source: Foods. 2025 Aug 28;14(17):3006. doi: 10.3390/foods14173006 (PMC12427750; doi:10.3390/foods14173006)
Supplement: Supplementary file 1 [file foods-14-03006-s001.zip › foods-3777521-supplementary.pdf]

Supplementary Information

Dissipation behavior and risk assessment of three pesticide residues under combined application in greenhouse-grown cabbage

Caixia Sun [1,*], Liping Chen [2], Yuhong Liu [1], Weiran Zheng [1], Yumei Hua[3] and Qiaoyan Zhang [1]

[1] Institute of Agro-product Safety and Nutrition, Zhejiang Academy of Agricultural Sciences, Hangzhou 310021, China
[2] Huzhou Agricultural Science and Technology Development Center, Huzhou 313009, China
[3] College of resources and environment, Huazhong Agricultural University, Wuhan 430072, China
* Correspondence: suncx@zaas.ac.cn

Table S1. Residues of three pesticides in cabbage

Pesticide	Treatment	Pesticide residues at different time (mg/kg)							
		0 (2h)	1 d	3 d	5 d	7 d	14 d	21 d	28 d
Azoxystrobin	Recommended dosages	6.06 ± 0.51	5.68 ± 0.44	5.28 ± 0.38	4.92 ± 0.55	4.74 ± 0.38	4.67 ± 0.42	4.35 ± 0.36	2.39 ± 0.25
	Double dosages	16.77 ± 0.82	13.60 ± 0.72	13.39 ± 0.71	13.08 ± 0.87	11.27 ± 0.57	6.22 ± 0.53	5.8 ± 0.37	5.21 ± 0.36
Thiamethoxam	Recommended dosages	2.17 ± 0.23	1.60 ± 0.16	1.36 ± 0.17	1.09 ± 0.11	1.02 ± 0.12	0.92 ± 0.09	0.76 ± 0.06	0.53 ± 0.03
	Double dosages	7.70± 0.45	4.61 ± 0.33	3.64 ± 0.34	3.48 ± 0.25	3.19 ± 0.27	1.54 ± 0.12	1.41 ± 0.09	1.12 ± 0.07
Carbendazim	Recommended dosages	36.55 ± 1.59	35.75 ± 2.31	35.29 ± 2.29	34.81 ± 1.09	33.67 ± 2.25	30.04 ± 2.06	28.11 ± 1.99	18.52 ± 1.37
	Double dosages	129.97 ± 4.85	95.08 ± 5.21	94.82 ± 4.52	93.04 ± 3.42	90.96 ± 4.35	56.31 ± 3.15	47.21 ± 2.35	46.58 ± 2.74
